# Silicone matrices for controlled dexamethasone release: toward a better understanding of the underlying mass transport mechanisms

**DOI:** 10.1093/rb/rbad008

**Published:** 2023-02-07

**Authors:** Thitiphorn Rongthong, Adam Qnouch, Maria Maue Gehrke, Laurent Paccou, Paulo Oliveira, Florence Danede, Jeremy Verin, Christophe Vincent, Jean-Francois Willart, Florence Siepmann, Juergen Siepmann

**Affiliations:** Univ. Lille, INSERM, CHU Lille, U1008, F-59000 Lille, France; Univ. Lille, INSERM, CHU Lille, U1008, F-59000 Lille, France; Univ. Lille, INSERM, CHU Lille, U1008, F-59000 Lille, France; Univ. Lille, UMR CNRS 8207, UMET, F-59655 Villeneuve d’Ascq, France; Univ. Lille, UMR CNRS 8207, UMET, F-59655 Villeneuve d’Ascq, France; Univ. Lille, UMR CNRS 8207, UMET, F-59655 Villeneuve d’Ascq, France; Univ. Lille, INSERM, CHU Lille, U1008, F-59000 Lille, France; Univ. Lille, INSERM, CHU Lille, U1008, F-59000 Lille, France; Univ. Lille, UMR CNRS 8207, UMET, F-59655 Villeneuve d’Ascq, France; Univ. Lille, INSERM, CHU Lille, U1008, F-59000 Lille, France; Univ. Lille, INSERM, CHU Lille, U1008, F-59000 Lille, France

**Keywords:** silicone matrix, drug release mechanism, dexamethasone, dexamethasone phosphate, Raman imaging

## Abstract

Dexamethasone-loaded silicone matrices offer an interesting potential as innovative drug delivery systems, e.g. for the treatment of inner ear diseases or for pacemakers. Generally, very long drug release periods are targeted: several years/decades. This renders the development and optimization of novel drug products cumbersome: experimental feedback on the impact of the device design is obtained very slowly. A better understanding of the underlying mass transport mechanisms can help facilitating research in this field. A variety of silicone films were prepared in this study, loaded with amorphous or crystalline dexamethasone. Different polymorphic drug forms were investigated, the film thickness was altered and the drug optionally partially/completely exchanged by much more water-soluble dexamethasone ‘phosphate’. Drug release studies in artificial perilymph, scanning electron microscopy, optical microscopy, differential scanning calorimetry, X-ray diffraction and Raman imaging were used to elucidate the physical states of the drugs and polymer, and of the systems’ structure as well as dynamic changes thereof upon exposure to the release medium. Dexamethasone particles were initially homogeneously distributed throughout the systems. The hydrophobicity of the matrix former very much limits the amounts of water penetrating into the system, resulting in only partial drug dissolution. The mobile drug molecules diffuse out into the surrounding environment, due to concentration gradients. Interestingly, Raman imaging revealed that even very thin silicone layers (<20 µm) can effectively trap the drug for prolonged periods of time. The physical state of the drug (amorphous, crystalline) did not affect the resulting drug release kinetics to a noteworthy extent.

## Graphical Abstract

**Figure:**
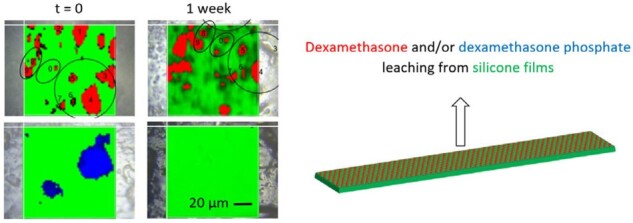

## Introduction

Local controlled drug delivery systems can very much improve the therapeutic efficacy of a drug treatment and reduce the risk of undesired toxic side effects [1–5]. Often, the drug is trapped within a polymeric matrix former, and different physico-chemical phenomena can be involved in the control of drug release [6–8]. Silicones offer an interesting potential as polymeric matrix formers, in particular for parenteral applications. For instance, drug-loaded vaginal rings have been developed for HIV prevention and hormonal contraception [9, 10]. ‘Norplant^®^’ is an example for a commercialized subcutaneous implant releasing the hormone levonorgestrel during 5 years for contraception [11]. Also, a variety of silicone-based drug delivery systems have been proposed for ‘ophthalmic’ applications, e.g. for the controlled release of timolol [12, 13]. Furthermore, silicone-based films have been developed for scar treatments [14] and for transdermal drug applications [15].

A broad range of drugs has been incorporated into silicone matrices for advanced medical therapies. The corticoid dexamethasone offers a particularly interesting therapeutic potential in this field, for instance to treat diseases of the inner ear (cochlea) [16–19], or to prolong the lifetime of pacemakers [20, 21]. In the latter case, the aim is to minimize fibrosis in the vicinity of the electrodes in order to keep the stimulation threshold values low. Clinical studies demonstrated the efficiency of this type of local controlled dexamethasone delivery in patients for at least 10 years [21]. In the case of the inner ear, the local controlled release of dexamethasone can provide multiple advantages for the treatment of patients suffering from severe deafness. The latest report of the World Health Organization (WHO) on hearing (2021) estimates that more than 1.5 billion people experience some degree of hearing loss [22]. In 2050, this number is expected to increase to ∼2.5 billion people. This will be around one-fourth of the world’s population. It has to be pointed out that hearing loss can have a severe impact on the quality of life of the patient and that it affects all ages [23, 24]. In the case of children, language development and education are often negatively affected. In the case of elderly, social isolation and loneliness are frequent consequences. Also, the patients are not able to live independently anymore, since they cannot communicate with others in a normal way. To allow such patients to hear again, one strategy is to place metal wires and electrodes into the inner ear: acoustic signals can be transformed into electrical ones, which are conducted by the wires into the cochlea to stimulate the hearing nerve. However, the inner ear is a tiny and highly sensitive organ: the placement of these ‘intracochlear’ implants is highly invasive and can lead to tissue trauma and inflammation. Furthermore, the efficiency of the transmission of electrical signals in the inner ear can substantially decrease with time, because the implant is a foreign body: the human organism isolates it by a fibrotic shell. The local delivery of dexamethasone can provide multiple key benefits, since this drug: (i) acts against inflammation, (ii) reduces fibrotic activity [25] and (iii) is expected to increase the lifetime expectancy of remaining hair cells (playing a key role for the hearing process), if delivered at low doses during long time periods. But the development of this type of advanced drug delivery systems is highly cumbersome, because very long release periods are targeted, e.g. several years or the lifetime of the patient. Drug product optimization based on series of trial-and-error experiments is extremely slow. For instance, the impact of the device design on long-term drug release is only obtained after years. This represents a crucial hurdle for innovative research in this highly promising field. It has to be pointed out that yet up to now, the reliable treatment of inner ear diseases and disorders with drugs is an unmet clinical need. To advance science in this field, a better understanding of the underlying mass transport mechanisms controlling drug release can be very helpful.

The physical and chemical phenomena, which can be involved in the control of the release rate of a drug out of a polymeric delivery system, can be rather complex [26–30]. A variety of phenomena might occur, including the penetration of water into the system, drug dissolution [31], the diffusion of dissolved drug out of the device [32], polymer swelling and dissolution [33], osmotic effects, local drops in micro pH [34], limited drug solubility effects ‘within’ the dosage form as well as in the surrounding bulk fluid/tissue, drug and/or polymer degradation and many others. Depending on the composition of the drug delivery system and its outer and inner structure, one or more processes can play a key role, while others are negligible. If several mass transport steps take place in a series and one of them is much slower than the others, this slowest step is rate-limiting for the entire drug release process. This can sometimes very much facilitate device optimization, e.g. if only drug diffusion is release rate controlling [35]. In certain systems, time-dependent changes in the conditions for drug release can very much complicate the underlying drug release mechanisms. For instance, polymer swelling [36], degradation [37] and/or dissolution can lead to time- and position-dependent drug diffusivities [38]. Also, crack formation might occur, altering drug mobility [39].

In the case of dexamethasone-loaded silicone matrices, diffusional mass transport has been reported to be of importance for drug release [16]. Also, the impact of the type of silicone and addition of different types and amounts of poly(ethylene glycol) on drug release has been studied [40]. However, many aspects still remain unclear, e.g. the impact of the physical state of the drug (e.g. amorphous vs. crystalline) on the resulting drug release kinetics. Also, advanced characterization techniques (like Raman imaging) have only very rarely been applied. The aim of this study was to get deeper insight into the mechanisms controlling drug release from silicone-based delivery systems. In particular: (i) different polymorphic forms of dexamethasone were to be investigated and compared to amorphous drug. (ii) The potential importance of drug batch-to-batch variability and of film thickness variations was to be studied. (iii) Raman imaging was to be used to monitor the fate of single drug particles upon exposure to aqueous fluids. (iv) The impact of adding much more hydrophilic dexamethasone ‘phosphate’ to the system on the spatial drug distribution in the silicone matrix as well as on drug leaching was to be investigated.

## Materials and methods

### Materials

To prepare silicone elastomers, the MED-4735 kit from NuSil Technology was used (Carpinteria, CA, USA). The following drugs were studied: dexamethasone (three batches with similar particle sizes in the lower micrometer range, but exhibiting different polymorphic forms: Batch 1: Form A, Batches 2 and 3: blends of Forms A and B) and dexamethasone sodium phosphate (dexamethasone phosphate) (Discovery Fine Chemicals, Dorset, UK). Acetonitrile was used as a solvent (HPLC grade; Fisher Scientific, Illkirch, France). For the preparation of artificial perilymph, the following compounds were used: potassium chloride, sodium chloride, calcium chloride dihydrate, magnesium sulfate tetrahydrate and 4-(2-hydroxyethyl) piperazine-1-ethanesulfonic acid (HEPES) (HEPES Pufferan) (Carl Roth, Lauterbourg, France).

### Preparation of drug-loaded films

To intimately blend the Parts A and B (∼5 g of each) of the NuSil MED-4735 kit, a two-roll mill (Chef Premier KMC 560/AT970A; Kenwood, Havant, UK) was used. First, the two parts were separately passed through the mill (10 times in each case). Then, the two parts were manually combined, and the blend was passed 10 times through the two-roll mill. This initiated polymer crosslinking. Afterwards, appropriate amounts of dexamethasone and/or dexamethasone phosphate were added and the mixtures were passed another 40 times through the mill to obtain homogenous films. The film thickness was ∼350 µm or 2 mm, as indicated. The drug loading was either ‘10% dexamethasone’ or ‘10% dexamethasone and 1% dexamethasone phosphate’, or ‘1% dexamethasone and 10% dexamethasone phosphate’, or ‘10% dexamethasone phosphate’, as indicated. Crosslinking was completed by a thermal treatment in an oven at 60°C for 24 h.

The dexamethasone powders were used as received in their respective crystalline forms. ‘Amorphous’ dexamethasone was obtained by ball milling crystalline drug (Batch 1: Form A) for 12 h at room temperature as follows: a high energy planetary mill (Pulverisette 7; Fritsch, Idar-Oberstein, Germany) was used, equipped with ZrO2 milling jars (43 cm^3^) with seven ZrO2 balls (φ = 15 mm). 1.1 g drug powder was placed into the mill. Thus, the ball weight: powder weight ratio was 75:1. The rotation speed of the solar disk was set to 400 rpm. Hence, the average acceleration of the balls was 5 *g* (*g* = acceleration of gravity). To limit the heating of the samples during the process, 15 min-milling periods (15 min) and 5 min-breaks were alternated.

In the case of dexamethasone ‘phosphate’, the powder (as received) was milled in a stainless-steel jar before it was incorporated into the silicone (as described above). A Retsch MM400 mill (Retsch, Haan, Germany) was used for this purpose. About 1 g drug was placed into a stainless-steel jar. The latter contained one stainless-steel ball. Milling was performed at 30 Hz for 3 min. Please note that the aim of this process was not the transformation of the drug into an amorphous form. This is why the milling conditions were much gentler compared to those used for the amorphization of dexamethasone.

### Drug release measurements

Drug release from 1 × 1 cm film pieces was measured in amber glass flasks, which were filled with 10 ml artificial perilymph. The latter was an aqueous solution of 5 mmol HEPES Pufferan, 2 mmol magnesium sulfate tetrahydrate, 145 mmol sodium chloride, 1.2 mmol calcium chloride dihydrate and 2.7 mmol potassium chloride. To assure agitation of the release medium, the amber flasks were placed into a horizontal shaker operating at 80 rpm (GFL 3033; Gesellschaft fuer Labortechnik, Burgwedel, Germany). The temperature was kept constant at 37°C. One milliliter of samples was withdrawn at predetermined time points. They were immediately replaced with fresh artificial perilymph (preheated at 37°C). To determine the drug concentrations in the withdrawn samples, HPLC–UV analysis was applied (λ = 220 nm): an Alliance e2695 apparatus was used (Waters Division, Milford, USA), which was equipped with an UV detector. Fifty microliters of samples were injected into a reverse phase column C18 (100 × 4.6 mm, Gemini 3 µm, 110 Å; Phenomenex, Le Pecq, France). The mobile phase was an acetonitrile:water 33:67 V:V mixture. The flow rate was set to 1.2 ml/min. The experiments were conducted in triplicate. Mean values ± standard deviations are reported.

### Monitoring of system swelling

To determine dynamic changes in the thickness and wet mass of the silicone films after exposure to artificial perilymph (37°C), a micrometer gauge (Digimatic Micrometer; Mitutoyo, Tokyo, Japan) as well as a Precisa 120A precision balance (Precisa, Dietikon, Switzerland) were used. Film samples were studied before and after exposure to artificial perilymph for different time periods. The conditions during exposure to the release medium were the same as for the *in vitro* drug release measurements (see ‘Drug release measurements’ section). Film samples were withdrawn at predetermined time points. Surface water was carefully removed using Kimtech tissue paper (Kimberly-Clark, TX, USA). The film thickness was measured using the micrometer gauge. The wet mass was measured using the precision balance. The experiments were conducted in triplicate. Mean values ± standard deviations are reported.

### Scanning electron microscopy

AJeol Field Emission Scanning Electron Microscope (JSM7800F, Tokyo, Japan) was used to obtain scanning electron microscopy pictures of drug powders. A ribbon carbon double-sided adhesive was used to fix the powder samples on the holders. A fine chrome layer was applied to cover the samples.

### Differential scanning calorimetry

A TA DSC Q10 apparatus (TA Instruments, Guyancourt, France) was used to record differential scanning calorimetry (DSC) thermograms in closed aluminum pans. The heating rate was set to 5°C/min. Temperature and enthalpy readings were calibrated using pure indium at the same heating rate. The calorimeter head was flushed with highly pure nitrogen gas during the measurements.

### X-ray diffraction

X-ray diffraction patterns were recorded with a Panalytical X’pert Pro diffractometer (transmission mode, λ Cu, Kα = 1.54 Å; PANalytical, Almelo, The Netherlands). An incident beam parabolic mirror was used. Samples were placed into Lindemann glass capillaries (Hilgenberg, Malsfeld, Germany) with a diameter of 1 mm. The capillaries were fixed on a spinning sample holder.

### Raman imaging

To monitor drug and silicone distribution in the films and dynamic changes thereof upon exposure to artificial perilymph, a Renishaw InVia Raman spectrometer was used. It was coupled to a Leica microscope. A ×50 long working distance Leica objective focused the 785 nm line, which was emitted from a Renishaw laser diode. Under the given experimental conditions, ∼200 µm^3^ sample volumes were analyzed at each XY position. The investigated spectral window ranged from 200 to 1800 cm^−1^. The spectral resolution was 2 cm^−1^. The classical sequential ‘point by point’ method was used to perform Raman mapping, scanning ‘100 × 100 µm^2^’ up to ‘500 × 500 µm^2^’ areas (with 1–5 µm distances between points). Six hundred up to 10 000 spectra were collected. The acquisition time ranged between 1 and 3 s. The direct classical least square (DCLS) method was used for the fitting of each measured sample spectrum to a linear combination of the Raman spectra of the reference spectra of the pure components. Films were studied before and after exposure to artificial perilymph. In the latter cases, the samples were treated under the same conditions as for the drug release measurements (see ‘Drug release measurements’ section). Film samples were withdrawn at predetermined time points. Kimtech tissue paper was used to remove surface water prior to the measurements.

## Results and discussion

The aim of this study was to better understand the underlying mass transport mechanisms in dexamethasone-loaded silicone matrices, which provide an interesting potential for a range of applications, e.g. allowing for innovative inner ear treatments or prolonging the lifetime of pacemakers. Critical key features to be studied included the physical states of the drug and polymer, the system structure/dimensions as well as dynamic changes thereof upon exposure to aqueous media.

### Crystalline vs. amorphous dexamethasone

The physical state of a drug might significantly affect its solubility in water and, thus, the rates at which it dissolves and at which it is released from a pharmaceutical dosage form. For instance, if the drug is distributed throughout a polymeric matrix in the form of drug ‘particles’, it first has to dissolve in order to become mobile and diffuse out of the system (due to concentration gradients). If the solubility of the drug and/or the amounts of water available for drug dissolution ‘within’ the system are limited, this can very much slow down drug release. Silicone matrices used for long-term controlled drug delivery are generally hydrophobic. Thus, only minor amounts of water can penetrate into the device and drug saturation effects can be crucial. Importantly, the solubility of a drug does not only depend on its ‘chemical’ structure but also potentially on its ‘physical’ state: a drug in an ‘amorphous’ form might exhibit a much higher aqueous solubility than in a crystalline state. Also, different polymorphic forms of the same drug might exhibit different solubility. Dexamethasone is known to form at least two different polymorphs: Form A and Form B [41].

**Figure 1.**
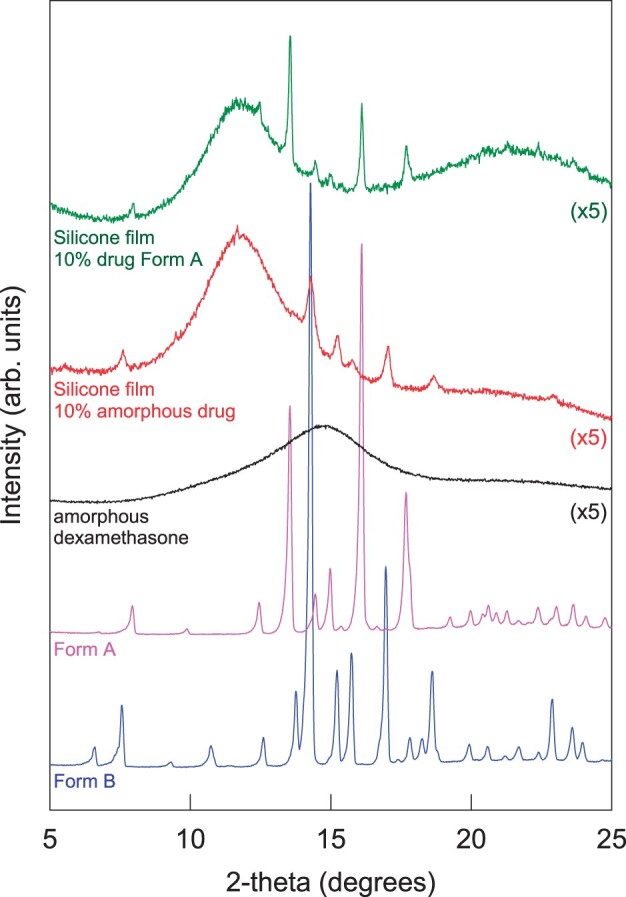
X-ray diffraction patterns of dexamethasone powders exhibiting different physical states and of silicone films prepared with amorphous or crystalline (Form A) drug.

**Figure 2.**
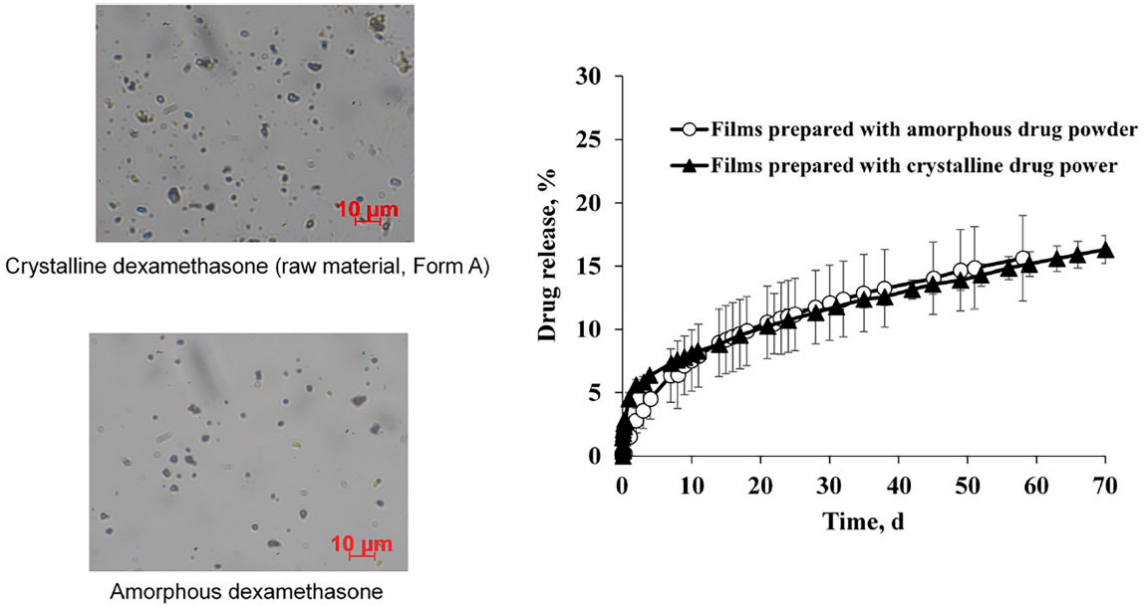
Left-hand side: optical microscopy pictures of crystalline (Form A) and amorphous drug powders. Right-hand side: dexamethasone release from silicone films prepared with crystalline (Form A) or amorphous drug in artificial perilymph at 37°C.

**Figure 3.**
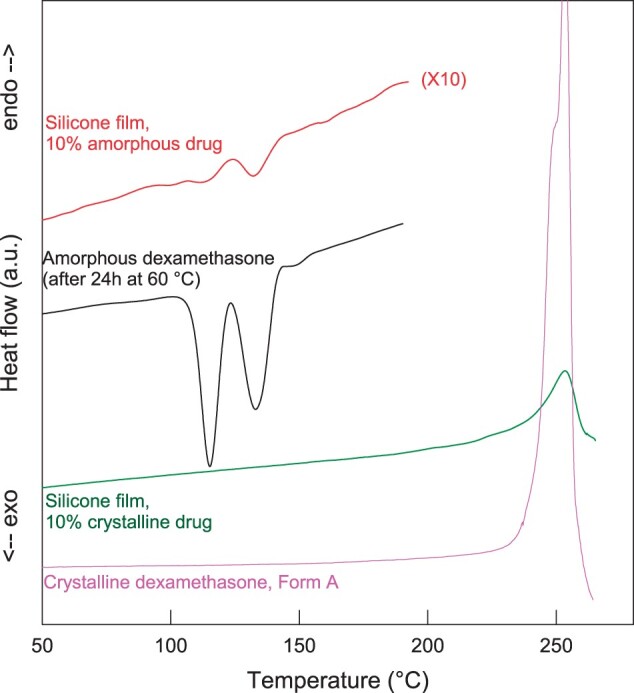
DSC thermograms of silicone films prepared with amorphous or crystalline (Form A) dexamethasone powder. For reasons of comparison, also the DSC thermograms of crystalline dexamethasone (Form A) and amorphous drug powder, which had been heated to 60°C for 24 h (=treatment to assure complete silicone cross-linking in films) prior to the measurement are shown.

In this study, three batches of dexamethasone were investigated: all were obtained from the same supplier, had similar particle sizes (in the lower micrometer range) and were crystalline. Batch 1 was the polymorphic Form A of dexamethasone. ‘Amorphous’ drug powder was obtained by milling samples of this batch for 12 h at room temperature in a high energy planetary mill. The pink and black curves in Fig. 1 show the X-ray diffraction patterns of the respective powders before and after milling. Optical microscopy pictures of samples are shown on the left-hand side of Fig. 2. The drug particle size did not substantially change upon milling (the aim was to alter the inner particle structure, not its size). Using the two types of powders, dexamethasone-loaded silicone films were prepared (10% w/w drug content). The green and red curves in Fig. 1 show the X-ray diffraction patterns of these films. As it can be seen, the polymorphic form of the crystalline dexamethasone (Form A) did not change during film manufacturing: sharp diffraction peaks are located at the same angles for the green and pink curves. However, the silicone films prepared with amorphous dexamethasone exhibited also sharp diffraction peaks (red curve). The latter cannot be attributed to the silicone (which was amorphous). Furthermore, the peak positions did not correspond to those of crystalline dexamethasone ‘Form A’ (which was used for film preparation, pink curve in Fig. 1). But they corresponded to the diffraction peaks of crystalline dexamethasone ‘Form B’ (blue curve in Fig. 1). This indicates that the amorphous dexamethasone used for film preparation was at least partially transformed into the crystalline Form B during manufacturing and/or film storage prior to the X-ray measurements. The DSC thermograms shown in Fig. 3 indicate that this solid-state transformation was not complete: the behavior of a silicone film prepared with 10% amorphous dexamethasone upon heating is illustrated (red curve). For reasons of comparison, also the thermogram of amorphous drug is shown (black curve). The amorphous drug had been heated to 60°C for 24 h prior to the measurement in order to mimic the heat treatment of the films to assure complete silicone cross-linking. In both cases, multiple events were visible, likely indicating surface and bulk recrystallization [41]. The green curve in Fig. 3 shows the thermal behavior of a silicone film prepared with dexamethasone Form A crystals: no signs for physical state transformations are visible until the drug melts at ∼250°C (corresponding to the endothermic event observed with dexamethasone polymorph Form A reference substance, pink curve).

Importantly, the above described transformations of the physical state of the drug did not affect the resulting dexamethasone release kinetics from silicone films to a noteworthy extent, as it can be seen in Fig. 2: the drug release patterns were similar from films prepared with crystalline dexamethasone Form A (filled triangles) and from films prepared with amorphous drug (open circles) (which at least partially recrystallized into the polymorphic Form B). This absence of any major effect on drug release might be explained by the fact that: (i) the differences in drug solubility of the respective forms are only minor and/or that (ii) the less soluble form relatively rapidly reprecipitates in the films (upon dissolution of the more soluble form) during drug release. It was beyond the scope of this study to investigate this aspect in more detail. From a practical point of view, it is most important that this type of formulation can be expected to be rather robust (‘forgiving’) with respect to the physical state of the drug in the formulation.

### Importance of drug batch-to-batch variability and film thickness

**Figure 4.**
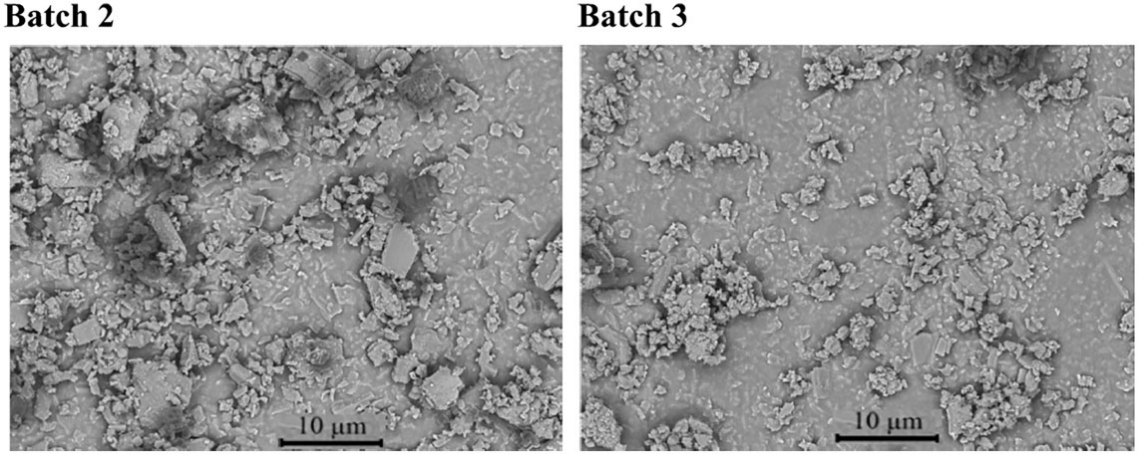
Scanning electron microscopy pictures of dexamethasone powder samples (as received): drug batches 2 and 3.

**Figure 5.**
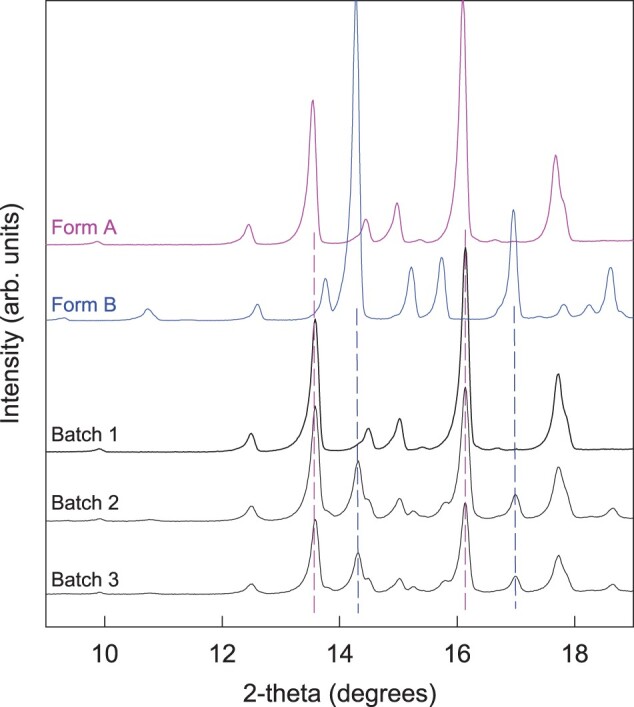
X-ray diffraction patterns of the investigated three batches of dexamethasone powders (as received), exhibiting similar particle sizes in the lower micrometer range, but different crystalline forms. For reasons of comparison, also the X-ray diffraction patterns of crystalline dexamethasone reference powders (polymorphs: Form A and Form B) are shown.

To evaluate the potential sensitivity of the key properties of dexamethasone-loaded silicone matrices (drug release and swelling behavior) to batch-to-batch variability of the drug raw material and variations in the film thickness, different types of films were prepared: two drug batches (Batches 2 and 3) were used for their preparation, and the film thickness was adjusted to ∼0.35 vs. 2 mm. The optical microscopy pictures in Fig. 4 show that there were no substantial differences in the particle sizes of the two drug batches (being in the lower micrometer size range). Interestingly, the two drug batches were blends of the two polymorphs: dexamethasone Form A and Form B (Form A being dominant). This is in contrast to the dexamethasone Batch 1 (which was pure Form A). Figure 5 shows the X-ray diffraction patterns of the three-drug batches, together with the reference patterns of dexamethasone Form A and Form B. As it can be seen, the peaks of Batch 1 correspond well to the peaks of dexamethasone Form A, whereas the peaks visible in drug batches 2 and 3 correspond to the peaks of both polymorphic forms: A and B (the dashed pink and blue lines are intended to help comparing key peaks of the two polymorphic forms).

**Figure 6.**
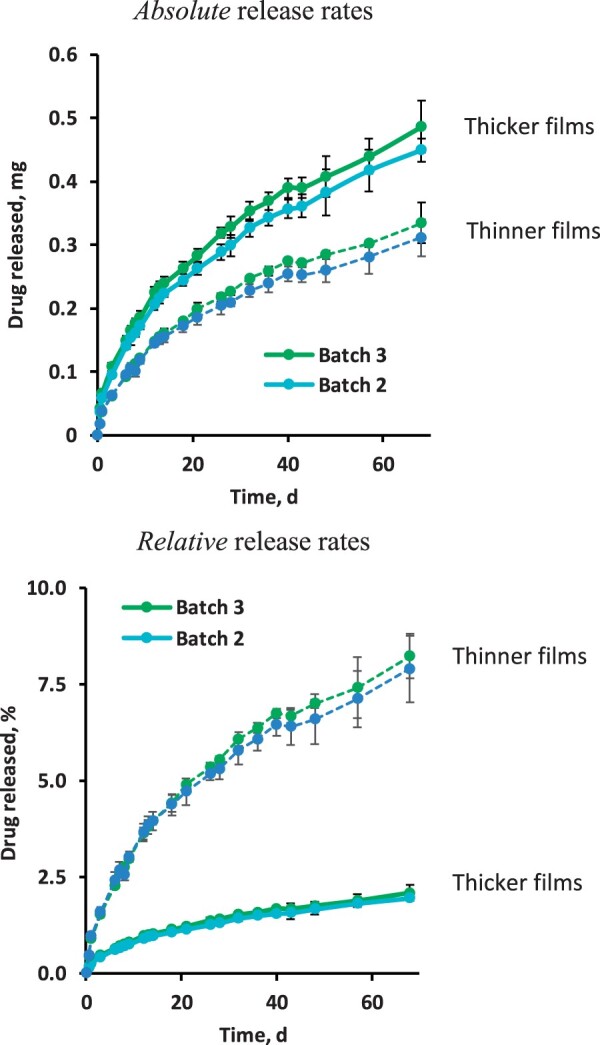
Dexamethasone is released from silicone films in artificial perilymph. Two different batches of dexamethasone raw material (Batches 2 and 3) were used for film preparation. ‘Thinner’ (∼350 µm, dashed curves) and ‘thicker’ (∼2 mm, solid curves) films were studied. At the top, the cumulative absolute amounts of drug release are shown, at the bottom the respective cumulative relative amounts of drug.

The experimentally measured drug release kinetics from silicone films loaded with 10% drug, prepared with drug batches 2 and 3, are shown in Fig. 6. At the top, the observed ‘absolute’ drug release rates are illustrated, at the bottom the respective ‘relative’ drug release rates. ‘Thinner’ (∼350 µm, dotted curves) and ‘thicker’ films (∼2 mm, solid curves) were studied. Clearly, the drug batch had no noteworthy impact on the resulting drug release kinetics, irrespective of the film thickness. Comparing the solid and dashed curves in the upper diagram in Fig. 6, it can be seen that the ‘absolute’ drug release rates were higher for ‘thicker’ than for ‘thinner’ films. This can be explained by the higher surface area available for diffusion (2.8 vs. 2.1 cm^2^). In contrast, the ‘relative’ drug release rates were lower for the ‘thicker’ films than for the ‘thinner’ films (solid vs. dashed curves in the bottom diagram in Fig. 6). This is because of the different 100% reference values (total drug loadings) of the film samples and the longer diffusion pathways for drug located in the center of thicker films. Please note that the films shown in Fig. 2 were even thinner than 350 µm, explaining the higher relative drug release rates compared to Fig. 6.

**Figure 7.**
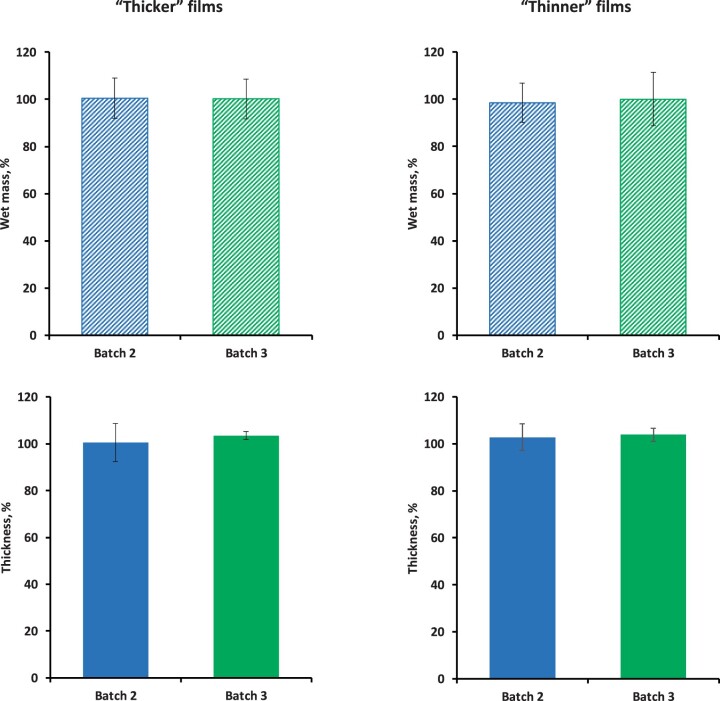
Absence of noteworthy film swelling or shrinking upon exposure to artificial perilymph: Wet mass (%) and thickness (%) of the films after 68-day exposure at 37°C. The 100% reference values are the initial sample mass and thickness. On the *x*-axes, the drug batches used for film preparation are indicated.

Figure 7 shows the swelling behavior of the ‘thinner’ and ‘thicker’ silicone films loaded with 10% dexamethasone, prepared with drug batch 2 or 3. The films were exposed to artificial perilymph for 68 days at 37°C. The top row illustrates the wet mass (%), the bottom row the film thickness (%). The 100% reference values are the film mass and thickness before exposure to the release medium. Clearly, there were no signs for noteworthy film swelling or shrinkage, irrespective of the initial film thickness and drug batch. From a practical point of view, this is a crucial aspect: for instance, if the drug delivery system is placed into a tiny and highly sensitive organ like the inner ear, significant device swelling causes serious damage.

### Drug distribution within the silicone matrices

**Figure 8.**
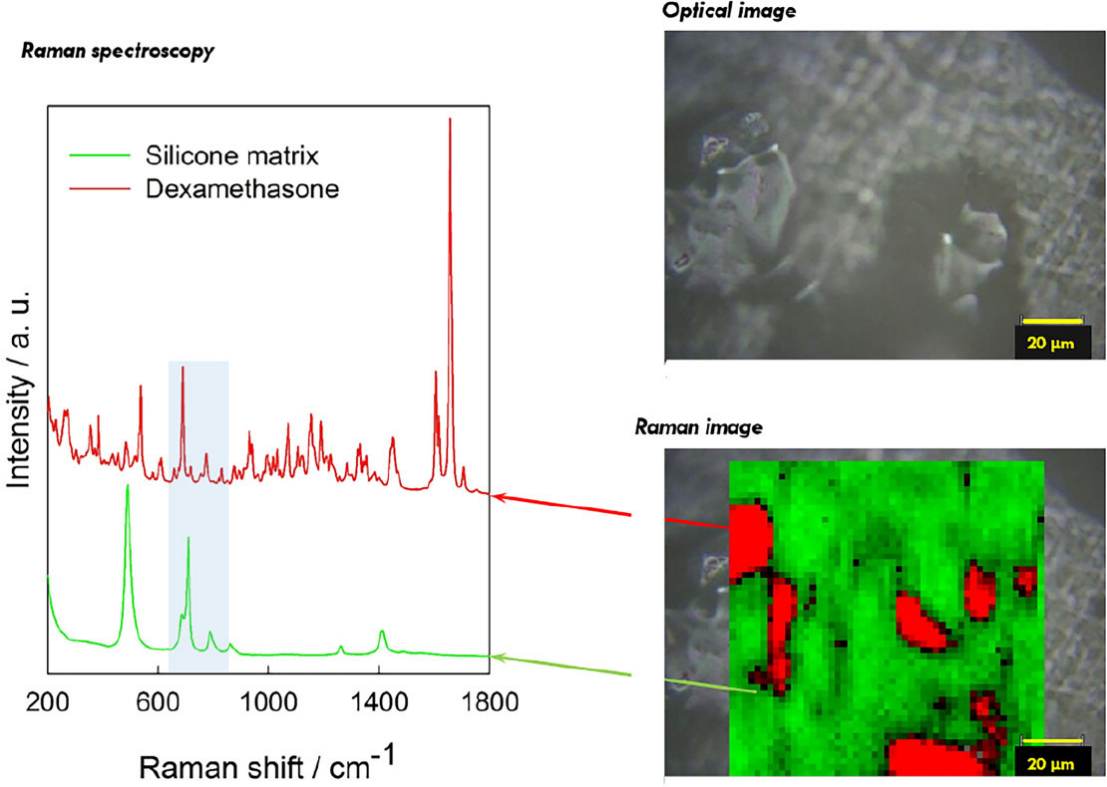
Left-hand side: Raman spectra of silicone and dexamethasone (Form A). Right-hand side: optical microscopy picture and Raman image of the surface of a silicone film loaded with 10% dexamethasone (prepared with crystalline drug, Batch 1: Form A) before exposure to the release medium. Silicone-rich regions are marked in green, dexamethasone-rich regions in red (false colors).

To better understand the structure of the investigated dexamethasone-loaded silicone matrices (as well as potential changes thereof during drug release), Raman imaging was applied. The diagram on the left-hand side of Fig. 8 shows the Raman spectra of dexamethasone (Form A) and silicone. As it can be seen, multiple Raman bands are located at different frequencies, allowing to distinguish between the two substances.

On the right-hand side of Fig. 8, an optical microscopy picture (top) and the corresponding Raman image of the surface of a silicone film before exposure to the release medium are shown. The film was loaded with 10% dexamethasone (prepared with crystalline drug, Batch 1: Form A). It was divided into small pixels, and the Raman spectrum of each pixel was recorded. A linear combination of the spectra of dexamethasone and silicone was fitted to the spectra of each pixel using the DCLS method. This allowed identifying pixels that were rich in silicone (marked in green) and pixels that were rich in dexamethasone (marked in red) (false colors). As it can be seen, certain regions were particularly rich in drug. When comparing the optical microscopy picture and Raman image in Fig. 8, it seems that these drug-rich regions correspond to zones with dexamethasone crystals.

**Figure 9.**
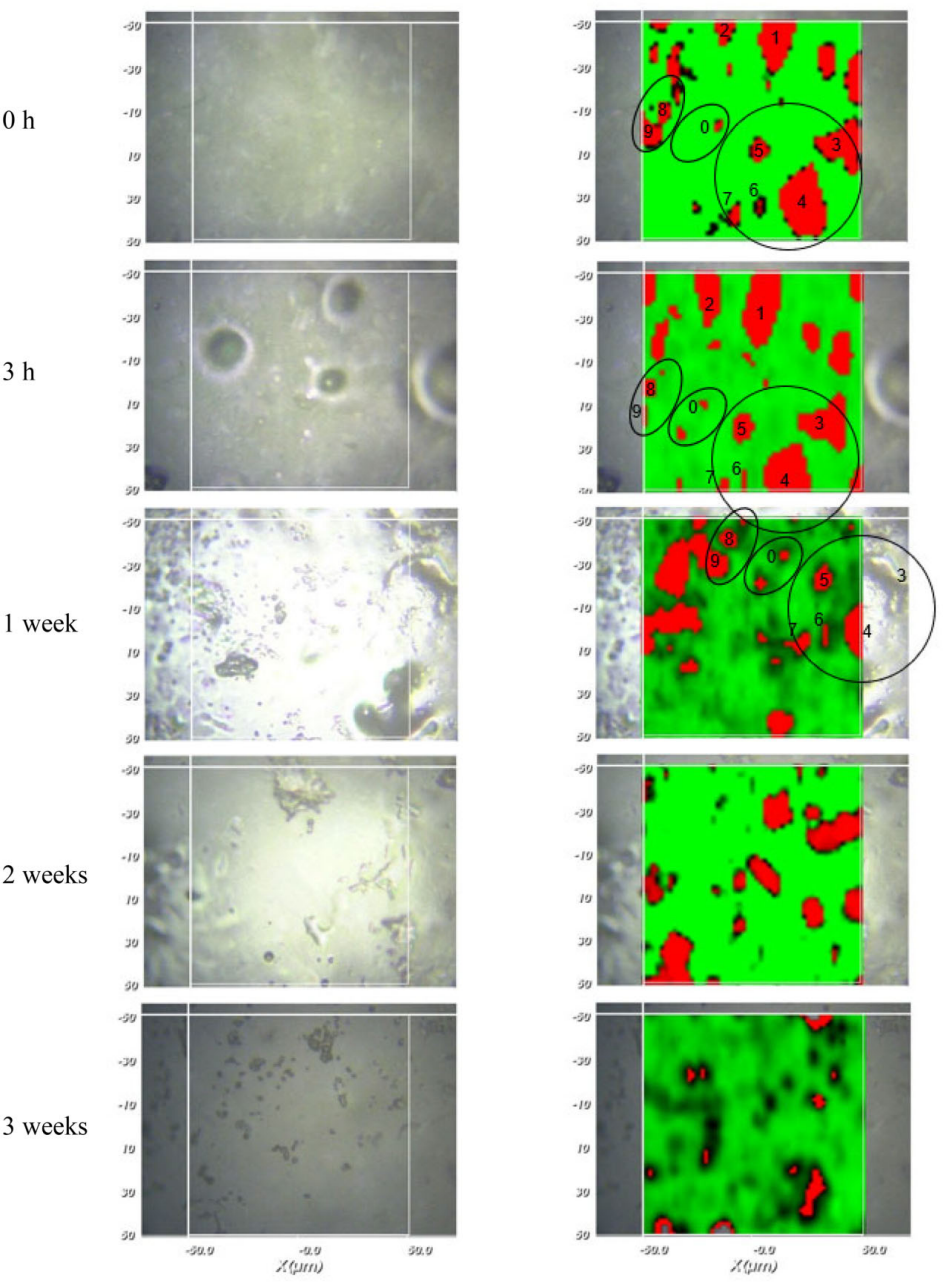
Optical microscopy pictures (left column) and Raman images (right column) of silicone films initially loaded with 10% dexamethasone (prepared with crystalline drug, Batch 1: Form A) after different exposure times to artificial perilymph at 37°C (indicated on the left-hand side). The circles highlight examples of specific locations, which are the same at different time points (the numbers correspond to specific drug particles). Silicone-rich regions are marked in green, dexamethasone-rich regions in red (false colors).

Figure 9 shows series of optical microscopy pictures and Raman images of a specific surface region of a silicone film initially loaded with 10% crystalline drug (Batch 1: Form A) before and after exposure to artificial perilymph at 37°C. The exposure times are indicated on the left-hand side. The aim was to analyze the same film zone at different time points. This was at least partially possible: the black circles in the Raman images highlight the same regions at different time points. The numbers correspond to specific drug particles. It has to be pointed out that the Raman measurements detected dexamethasone up to a depth of about 20–25 µm in this study. Thus, drug crystals visible in the top row of Fig. 8 (before exposure to the release medium) might be directly located at the film’s surface or might be separated from the surface by a thin silicone layer. This explains why not all dexamethasone particles disappear upon exposure to the artificial perilymph: only those with direct surface access (and, thus, being in direct contact with water) can rapidly dissolve. Looking at Fig. 9, it seems that the majority of the drug crystals did not have immediate surface access and remained within the film during the observation period. This is in good agreement with the very slow drug release observed from these films (discussed above) and consistent with recent findings on dexamethasone distribution within cylindrical, cochlear implants based on silicone [42]. It seems that the dexamethasone particles are initially homogeneously distributed throughout the polymeric matrices. Once in contact with the release medium, only very limited amounts of water penetrate into the system, because of the hydrophobic nature of the silicone. These limited amounts of water partially dissolve the drug particles. Once dissolved, individualized dexamethasone molecules diffuse out of the system, due to concentration gradients.

**Figure 10.**
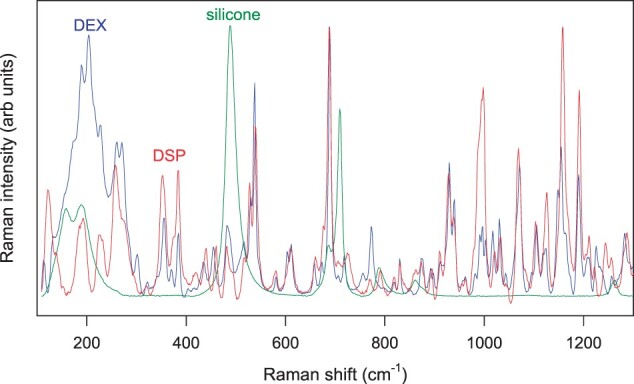
Raman spectra of silicone, dexamethasone (DEX) and dexamethasone phosphate (DSP).

**Figure 11.**
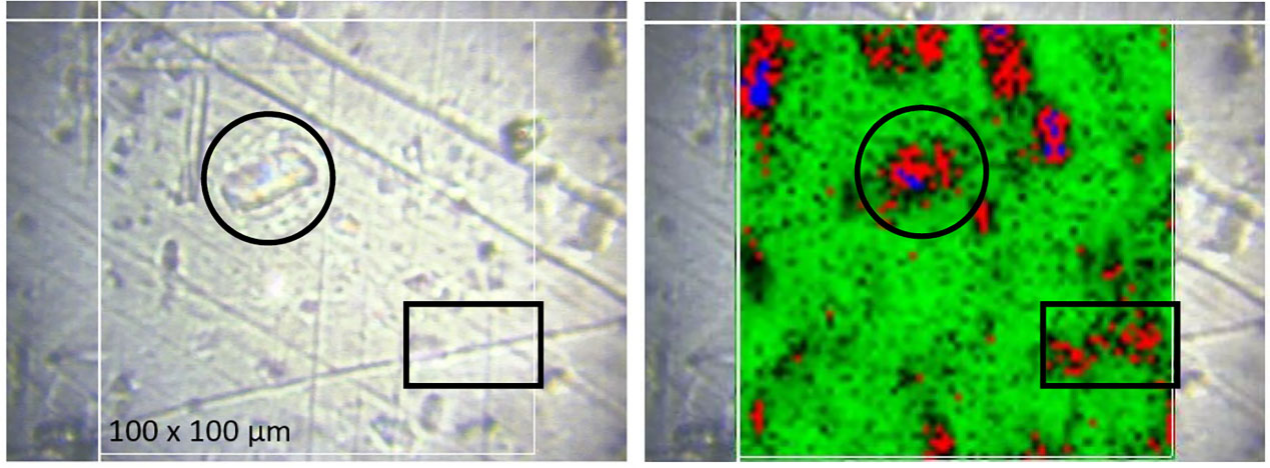
Optical microscopy picture (left) and Raman image (right) of the surface of a silicone film loaded with 10% dexamethasone and 1% dexamethasone phosphate (both crystalline, dexamethasone Batch 1: Form A) before exposure to the release medium. Silicone-rich regions are marked in green, dexamethasone-rich regions in red and dexamethasone phosphate-rich regions in blue (false colors).

**Figure 12.**
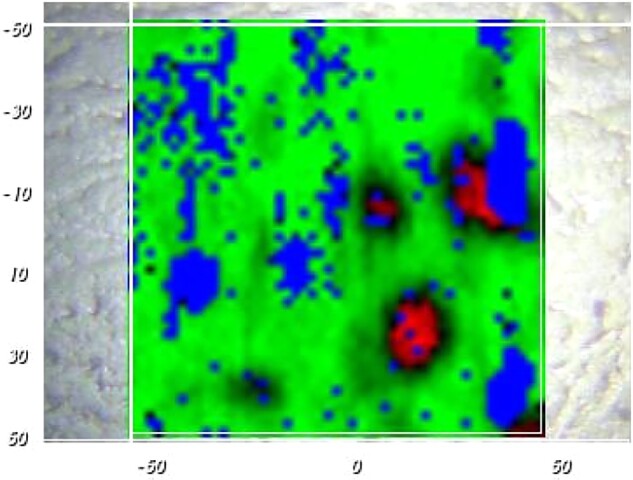
Raman image of the surface of a silicone film loaded with 1% dexamethasone (Batch 1: Form A) and 10% dexamethasone phosphate before exposure to the release medium. Silicone is marked in green, dexamethasone in red and dexamethasone phosphate in blue (false colors). scales are in µm.

**Figure 13.**
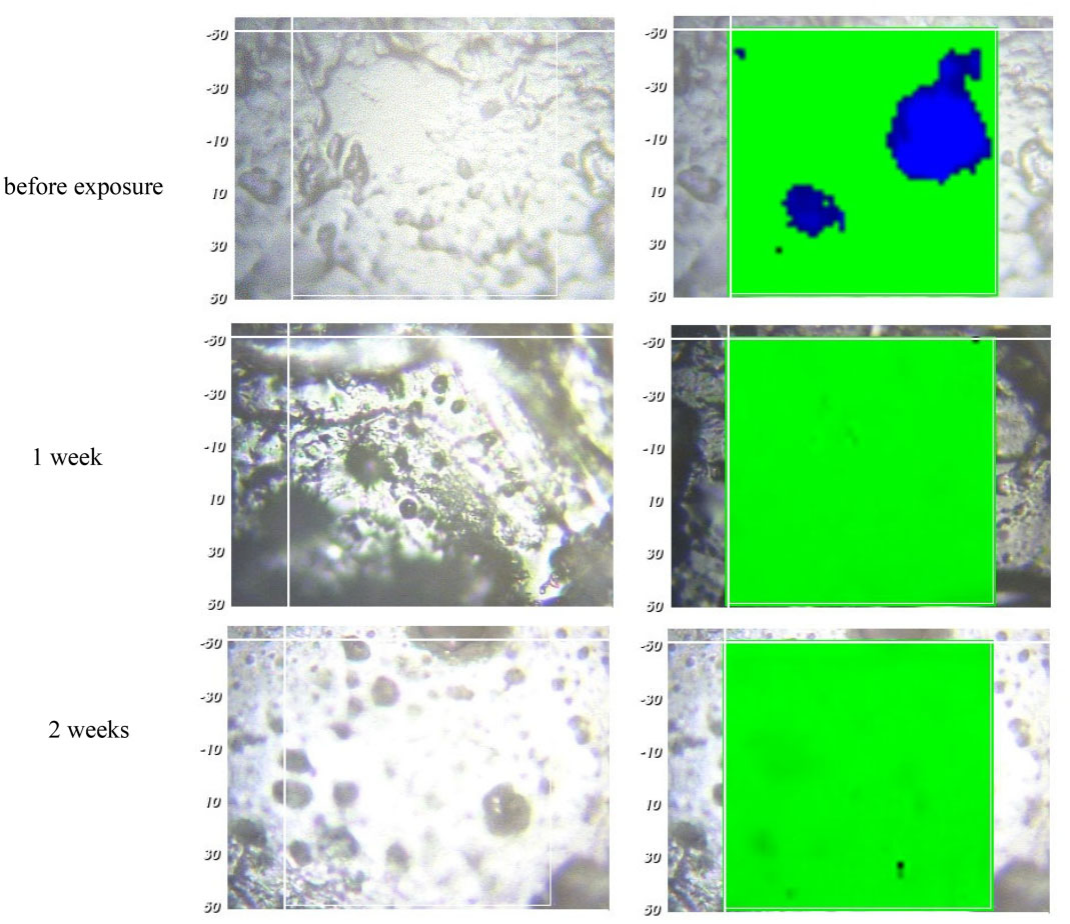
Optical microscopy pictures (left column) and Raman images (right column) of silicone films initially loaded with 10% dexamethasone phosphate before and after different exposure times to artificial perilymph at 37°C (as indicated on the left-hand side, scales are in µm). Silicone is marked in green, dexamethasone phosphate in blue (false colors).

In order to increase the resulting drug release rate, especially at early time points (‘burst effect’), the addition of much more water-soluble dexamethasone ‘phosphate’ has been proposed [19]. Importantly, also the Raman spectrum of dexamethasone ‘phosphate’ shows distinct differences in its bands compared to dexamethasone and silicone (Fig. 10). Thus, Raman imaging allows distinguishing between the three compounds and identifying pixels, which are particularly rich in silicone, dexamethasone or dexamethasone ‘phosphate’. Figure 11 shows an optical microscopy picture and a Raman image of a silicone film loaded with 10% dexamethasone and 1% dexamethasone ‘phosphate’ before exposure to the release medium. Both drugs were crystalline (dexamethasone Batch 1: Form A was used for the preparation). Silicone-rich regions are marked in green, dexamethasone-rich regions in red and dexamethasone ‘phosphate’-rich regions in blue (false colors). Interestingly, the dexamethasone and dexamethasone phosphate particles seem to be located in the same regions. This can probably be explained by the fact that both types of drug particles do not have a high affinity toward the investigated silicone (nor to the compounds used for film preparation: Parts A and B of the MED-4735 kits). To reduce the contact surface area ‘drug–silicone’, the drug particles partially agglomerated. This is important information for the underlying drug release mechanism: once an interconnected drug particle network gets access to the film’s surface (e.g. via a water-filled pore), it can be expected that all ‘connected’ particles can rather rapidly diffuse out into the surrounding bulk fluid. Figure 12 shows a Raman image of a silicone film loaded with 1% dexamethasone (Batch 1: Form A) and 10% dexamethasone phosphate before exposure to the release medium. So, the dexamethasone:dexamethasone phosphate ratio was ‘inversed’ compared to the film illustrated in Fig. 11 (1:10 instead of 10:1). The dominance of dexamethasone phosphate compared to dexamethasone was clearly visible in this outer film layer. This can be expected to have important consequences for the resulting drug release kinetics: dexamethasone phosphate being much more soluble in water than dexamethasone, likely attracts more water into the system upon exposure to the artificial perilymph. This is consistent with the recently reported increase of the ‘initial burst release’ from dexamethasone-loaded silicone films upon addition of dexamethasone ‘phosphate’ [19]. The Raman images in Fig. 13 further confirm this hypothesis: a silicone film initially loaded with 10% dexamethasone ‘phosphate’ is shown ‘before and after’ different exposure times to artificial perilymph at 37°C (as well as the corresponding optical microscopy pictures). Before exposure to the release medium, large drug clusters can be seen, even larger than those observed with dexamethasone (e.g. Figs 8 and 9). This might be explained by the higher hydrophilicity of dexamethasone ‘phosphate’ compared to dexamethasone, the silicone being hydrophobic. Interestingly, after 1-week exposure to the artificial perilymph, no drug was detectable anymore. This is in contrast to the observed behavior of dexamethasone (which could still be detected in considerable amounts at this time point in surface-near regions, Fig. 9). This can be attributed to the higher hydrophilicity of this drug, attracting more water into the system, and resulting in higher absolute and relative drug release rates [19].

## Conclusion

Limited drug solubility and diffusional mass transport seem to play key roles for the control of dexamethasone release from silicone matrices. Drug particles are initially homogeneously distributed throughout the system. Upon contact with aqueous fluids, only very limited amounts of water penetrate into the device, due to the hydrophobicity of the matrix former. The low amounts of water partially dissolve the drug particles. Once dissolved, the drug diffuses out of the system, due to concentration gradients. Interestingly, the physical state of the dexamethasone particles (crystalline vs. amorphous, type of polymorphic form) did not significantly impact drug release in the investigated systems. Raman imaging allows monitoring the fate of ‘individual’ drug particles over time, providing deeper insight into the underlying mass transport mechanisms. Importantly, even very thin silicone layers (<20 µm), separating dexamethasone particles from aqueous bulk fluid, can effectively trap the drug during long periods of time. Replacing dexamethasone by much more hydrophilic dexamethasone ‘phosphate’ leads to significantly faster drug depletion.
